# Genomics-assisted breeding for boosting crop improvement in pigeonpea (*Cajanus cajan*)

**DOI:** 10.3389/fpls.2015.00050

**Published:** 2015-02-17

**Authors:** Lekha Pazhamala, Rachit K. Saxena, Vikas K. Singh, C. V. Sameerkumar, Vinay Kumar, Pallavi Sinha, Kishan Patel, Jimmy Obala, Seleman R. Kaoneka, P. Tongoona, Hussein A. Shimelis, N. V. P. R. Gangarao, Damaris Odeny, Abhishek Rathore, P. S. Dharmaraj, K. N. Yamini, Rajeev K. Varshney

**Affiliations:** ^1^International Crops Research Institute for the Semi-Arid TropicsPatancheru, India; ^2^African Centre for Crop Improvement, School of Agricultural, Earth and Environmental Sciences, University of KwaZulu-NatalScottsville, South Africa; ^3^International Crops Research Institute for the Semi-Arid TropicsNairobi, Kenya; ^4^Agricultural Research Station, University of Agricultural SciencesGulbarga, India; ^5^Department of Agricultural Biotechnology, Acharya N. G. Ranga Agricultural UniversityHyderabad, India; ^6^School of Plant Biology and Institute of Agriculture, The University of Western AustraliaCrawley, WA, Australia

**Keywords:** pigeonpea, genetic variability, genomic resources, genomics-assisted breeding, marker assisted selection, genomic selection

## Abstract

Pigeonpea is an important pulse crop grown predominantly in the tropical and sub-tropical regions of the world. Although pigeonpea growing area has considerably increased, yield has remained stagnant for the last six decades mainly due to the exposure of the crop to various biotic and abiotic constraints. In addition, low level of genetic variability and limited genomic resources have been serious impediments to pigeonpea crop improvement through modern breeding approaches. In recent years, however, due to the availability of next generation sequencing and high-throughput genotyping technologies, the scenario has changed tremendously. The reduced sequencing costs resulting in the decoding of the pigeonpea genome has led to the development of various genomic resources including molecular markers, transcript sequences and comprehensive genetic maps. Mapping of some important traits including resistance to *Fusarium* wilt and sterility mosaic disease, fertility restoration, determinacy with other agronomically important traits have paved the way for applying genomics-assisted breeding (GAB) through marker assisted selection as well as genomic selection (GS). This would accelerate the development and improvement of both varieties and hybrids in pigeonpea. Particularly for hybrid breeding programme, mitochondrial genomes of cytoplasmic male sterile (CMS) lines, maintainers and hybrids have been sequenced to identify genes responsible for cytoplasmic male sterility. Furthermore, several diagnostic molecular markers have been developed to assess the purity of commercial hybrids. In summary, pigeonpea has become a genomic resources-rich crop and efforts have already been initiated to integrate these resources in pigeonpea breeding.

## Introduction

Pigeonpea [*Cajanus cajan* (L.) Millspaugh] is the sixth most important grain legume of the world. The crop is predominantly grown in Asia, Africa and the Caribbean Islands, India being the largest producer with 2.86 million tons (FAOSTAT, 2012). Pigeonpea has multiple uses, for instance, the split peas are rich source of protein (20–23%) and form an excellent combination with cereals for a balanced human diet. Additionally, it improves soil fertility by fixing atmospheric nitrogen and reducing soil erosion. Pigeonpea has a genome size of 833.07 Mb and is the first non-industrial food legume crop for which draft genome sequence has been developed (Varshney et al., 2012a). It is an often cross-pollinated diploid (2n = 2x = 22) crop and the natural out-crossing ability has been utilized to develop an efficient cytoplasmic genetic male sterility (CGMS) based hybrid system in pigeonpea (Saxena et al., 2010a; Varshney et al., 2010).

Over the last six decades, pigeonpea productivity has remained stagnant at around 780 kg/ha, mainly due to the exposure of the crop to various biotic and abiotic stresses. Besides, it is mostly grown in marginal environments with limited inputs and inefficient management practices (Varshney et al., 2012b).

The major challenge for pigeonpea improvement is increasing the productivity at the same time reducing the yield losses due to various biotic and abiotic stresses under changing climate scenario. This emphasizes the need for developing high yielding varieties with resistance to multiple stresses to survive the challenges of the marginal environments. Genomics-assisted breeding (GAB) can help the breeders to select suitable parents for different crossing programs so as to have novel combinations leading to elite breeding lines. However, inadequate genomic resources coupled with narrow genetic base in cultivated gene pool caused serious impediment to applying GAB for pigeonpea improvement (Varshney et al., 2010). To overcome this, several research groups were engaged in developing genomic resources during the last few years. As a result large number of molecular markers including simple sequence repeat (SSR), diversity array technology (DArT), single feature polymorphism (SFPs) and single nucleotide polymorphisms (SNPs) were developed. Additionally, various genetic resources including inter-specific and intra-specific mapping populations were also developed (see Bohra et al., 2014a). This review article highlights the genomics efforts made, exploring the future possibilities and potential challenges of GAB for pigeonpea improvement.

## Genetic resources

Pigeonpea is one of the ancient crop originated in India (van der Maesen, 1990; Kassa et al., 2012; Saxena et al., 2014), from where it is believed to have taken to Africa before 2000 BC (van der Maesen, 1990; Songok et al., 2010). The genus *Cajanus* includes 32 species and belongs to the sub-tribe *Cajaninae* (van der Maesen, 1990; Bohra et al., 2010), of which *C. cajan* (2n = 2x = 22) represents the domesticated species (Rao et al., 2003; Bohra et al., 2010). *C. cajan* occupies the primary gene pool, whereas the wild progenitors are placed in the secondary and the tertiary gene pool based on their crossibility with the cultivated species (see Bohra et al., 2010).

Pigeonpea germplasm is represented by a total of 13,771 accessions deposited at the genebank in ICRISAT, India (Gowda et al., 2013), 11,221 accessions collected at National Bureau of Plant Genetic Resources (NBPGR), India (Singh et al., 2014), 4,116 accessions at U.S. Department of Agriculture (USDA), USA and 1,288 accessions at Kenya Agricultural Research Institute's National Genebank of Kenya (KARI-NGBK), Kenya (Singh et al., 2013). These are the reservoirs of genetic resources for the present and future pigeonpea improvement programmes. Utilization of these genetic resources for pigeonpea improvement is very limited and majority of diversity existing in the germplasm remained unexplored (Majumder and Singh, 2005). To address these issues, ICRISAT has defined a core collection of 1,290 accessions and a mini core collection of 146 accessions (Gowda et al., 2013). These collections represent more than 80% of the diversity existing in the entire germplasm collection and are ideal resources for studying genetic diversity, population structure and association mapping (Reddy et al., 2005; Upadhyaya et al., 2006; Gowda et al., 2013). The ICRISAT genebank has maintained 555 accessions representing 67 wild species from six genera (Upadhyaya et al., 2011). This exclusive collection has been extensively screened to identify accessions (wild and cultivated) harboring valuable traits (Kumar et al., 2011; Sharma et al., 2013; Upadhyaya et al., 2013). Various accessions have been characterized for early maturity, large seed size, high pod number per plant, high seed protein content, high iron, high zinc, tolerance to salinity and water-logging (See Gowda et al., 2013). Many wild relatives have been identified to possess enormous potential to render desirable traits such as tolerance to abiotic stresses, resistance to pests and diseases, high protein content, photo-insensitivity, cleistogamy and cytoplasmic male sterility (Table 1).

**Table 1:** **Different wild species of pigeonpea useful for rendering valuable traits**.

**Important traits**		**Wild relatives**	**References**
Resistance to pests	Pod borer	*C. scarabaeoides*	Sharma et al., 1987
		*C. acutifolius*	Saxena and Sharma, 1990
		*C. platycarpus*	Sharma, 1995
		*C. reticulatus*	Rao et al., 2003
		*C. sericeus*	Kulkarni et al., 2003
		*C. albicans*	Sujana et al., 2008
	Pod fly	*C. reticulatus*	Sharma et al., 2009
		*C. acutifolius*	Mallikarjuna et al., 2007
			Sharma et al., 2003
	Bruchids	*C. scarabaeoides*	Jadhav et al., 2012
		*C. acutifolius*	Mallikarjuna et al., 2011
		*C. platycarpus*	
Resistance to diseases	Fusarium wilt	*C. platycarpus*	Mallikarjuna et al., 2011
	Sterility mosaic disease	*C. sericeus*	Akinola and Whiteman, 1975
		*C. albicans*	
		*C. volubilis*	Singh et al., 2005
		*C. lineatus*	
	Phytophthora blight	*C. platycarpus*	Mallikarjuna et al., 2005, 2006
		*C. sericeus*	
		*C. acutifolius*	Akinola and Whiteman, 1975
Grain quality	High protein content	*C. cajanifolius*	Akinola and Whiteman, 1975
		*C. sericeus*	
		*C. albicans*	Dalvi et al., 2008
		*C. lineatus*	
		*C. scarabaeoides*	
	High seed weight	*C. acutifolius*	Jadhav et al., 2012
Cytoplasmic male sterility	-	*C. cajanifolius*	Tikka et al., 1997
		*C. sericeus*	Saxena and Kumar, 2003
		*C. scarabaeoides*	
		*C. acutifolius*	Ariyanayagam et al., 1993, 1995
		*C. volubilis*	
		*C. platycarpus*	Wanjari et al., 2000
			Saxena et al., 2005
			Saxena et al., 2010a
			Mallikarjuna et al., 2011
Tolerance to abiotic stresses	Salinity	*C. platycarpus*	Subbarao et al., 1990
			Srivastava et al., 2006
	Drought	*C. sericeus*	Subbarao et al., 1990, 1991
		*C. albicans*	
		*C. lineatus*	Rao et al., 2003
		*C. scarabaeoides*	Srivastava et al., 2006
	Water-logging	*C. scarabaeoides*	Krishnamurthy et al., 2012
Plant type	Extra-early flowering maturity	*C. platycarpus*	Mallikarjuna and Moss, 1995
			Saxena, 2008
	Photo-insensitivity	*C. platycarpus*	Mallikarjuna and Moss, 1995
	Cleistogamy	*C. lineatus*	Saxena et al., 1992

The wild species are slowly diminishing with their habitat gradually shrinking and are likely to be extinct. Of late, the importance of these invaluable reservoirs of useful traits is being realized and efforts have been made to rescue them. In this scenario, genebanks will play a major role in conserving and utilizing them for the on-going and future crop improvement programmes. These wild species remained underutilized due to linkage drag and cross-incompatibility with the cultivated species. Due to this cross-incompatibility (post-zygotic or pre-zygotic barriers), only inter-specific derivatives could be made with wild species of the secondary gene pool. The wild relative, *C. platycarpus* of the tertiary gene pool having many desirable traits (Table 1), but could not be crossed with *C. cajan* by conventional techniques (Mallikarjuna and Moss, 1995; Mallikarjuna et al., 2005). In this regard, wide hybridization or the embryo-rescue technique has been found promising for overcoming the crossing barriers in pigeonpea (Mallikarjuna, 2003, 2007; Srikanth et al., 2014). Concerted efforts must be made for the introgression of novel genes from the secondary and tertiary gene pools for multiple stress resistance, unique agronomic traits (favorable growth habit, enhanced nutritional qualities, high yield potential) and broadening the genetic base of the cultivated gene pool. Thus, the existing germplasm should effectively be utilized for identifying and mining new sources of allelic variation associated with the agronomically beneficial traits (Gowda et al., 2013).

In addition, there is a need to develop specialized genetic resources for trait dissections. For instance, mapping populations are the pre-requisites for the development of genetic maps and identification of quantitative trait loci (QTLs) for the desired traits. In this respect, about 30 segregating mapping populations for different biotic and abiotic stresses were developed worldwide; the details of these populations are available in Varshney et al. (2010) and Bohra et al. (2014a). For instance, different F_2_ mapping populations have been developed for mapping biotic (resistance to Fusarium wilt, sterility mosaic disease and pod borer), abiotic stresses (drought tolerance), fertility restoration, plant type and earliness (Table 2). These mapping populations were used for identifying QTLs and for generating various inter- and intra-specific genetic maps.

**Table 2:** **Genetic resources available for mapping different traits in pigeonpea**.

**Trait**	**Type of population**	**Population**	**References**
*Fusarium* wilt resistance	Intra-specific F_2_	ICPB 2049 × ICPL 99050	Bohra et al., 2012
*Fusarium* wilt resistance	F_2_	GSl × ICPL 87119	Kotresh et al., 2006
		GS1 × ICP 8863	
Sterility mosaic disease resistance	Intra-specific F_2_	ICP 8863 × ICPL 20097	Gnanesh et al., 2011
		TTB 7 × ICP 7035	
Fertility restoration	F_2_	ICPA 2039 × ICPR 2447	Bohra et al., 2011
		ICPA 2043 × ICPR 3467	
		ICPA 2043 × ICPR 2671	
Pod borer	Inter-specific F_2_	ICPL 8755 × ICPL 227	Saxena et al., 2011
		ICPL 151 × ICPL 87	
		ICP 28 × ICPW 94	
Drought tolerance	Inter-specific F_2_	ICPL 8755 × ICPL 227	Saxena et al., 2011
		ICPL 151 × ICPL 87	
		ICP 28 × ICPW 94	
Determinacy	F_2_	ICPL 85010 × ICP 15774	Mir et al., 2014
Plant type	F_2_	TT44-4 × TDT2004-1	Dhanasekar et al., 2010
Plant height, number of primary and secondary branches, number of pods, days to maturity and days to flowering	F_2_	Pusa Dwarf × HDM04-1	Kumawat et al., 2012

Other than the above mentioned bi-parental mapping populations, multi-parent mapping populations such as multi-parent advanced generation inter-cross (MAGIC) and nested association mapping (NAM) populations are also being developed. Such mapping populations will not only be useful for high resolution genetic mapping but would also be an excellent breeding materials *per-se* to develop superior varieties based on phenotypic performance and could be utilized for different crossing programs to enhance the genetic base of cultivated gene pools in pigeonpea.

## Genomic resources

**Figure 1:**
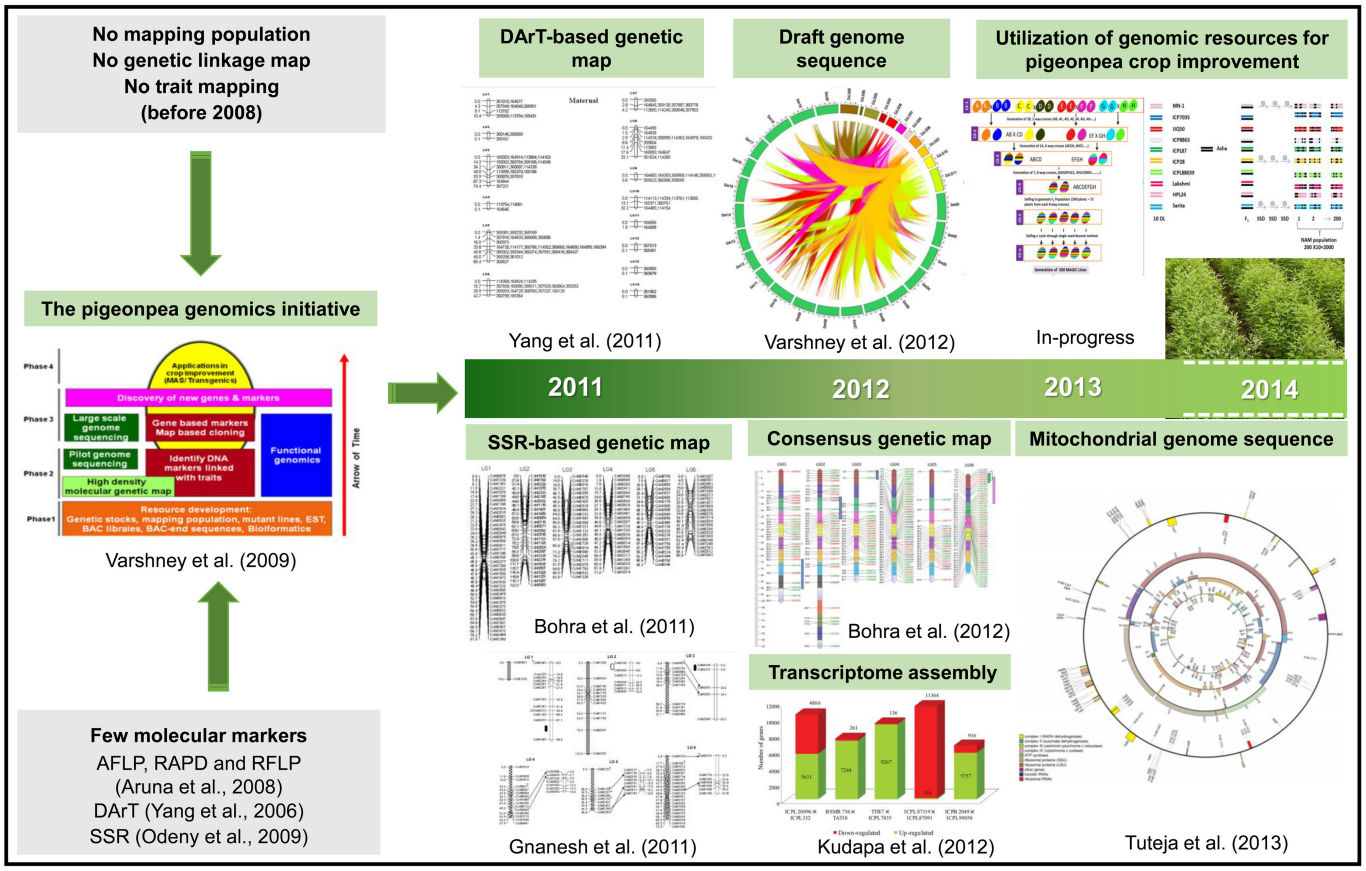
**A glimpse of the pigeonpea genomics research**. The figure depicts the present scenario in terms of major genomic resources developed during the last decade that would lead to pigeonpea crop improvement. There were very few markers and with no mapping population developed before Pigeonpea Genomics Initiative (PGI). Since the inception of PGI in 2006, progress were made in developing various genetic and genomic resources, including different inter- and intra-specific mapping populations, molecular markers, genetic maps and transcriptome assemblies. In 2012, the pigeonpea genome was decoded which has been a milestone in pigeonpea research. In the present scenario, efforts have been initiated to deploy genomic-assisted breeding for pigeonpea improvement.

In terms of genomic resources, limited number of SSR markers were available for pigeonpea (Varshney et al., 2009). Subsequently, only few genetic maps with poor marker density were developed (Varshney et al., 2010). However, in recent past significant efforts have been made to develop a range of genomic resources (Figure 1).

### Molecular markers

Molecular markers have been found very useful for molecular breeding, in enhancing the genetic gain and reducing the breeding cycles in many crop species (Varshney et al., 2014a). The first generation of molecular markers included restriction fragment length polymorphism (RFLP; Nadimpalli et al., 1993; Sivaramakrishnan et al., 1997; Lakshmi et al., 2000; Sivaramakrishnan et al., 2002), random amplified polymorphic DNA (RAPD; Ratnaparkhe et al., 1995; Lohithaswa et al., 2003; Choudhury et al., 2008; Malviya and Yadav, 2010) and amplified fragment length polymorphism (AFLP; Panguluri et al., 2005; Wasike et al., 2005; Aruna et al., 2008) have been developed and employed mainly for diversity studies. SSR markers, belonging to the second generation markers, were developed from the genome sequence (gSSRs), expressed sequence tags (ESTs-SSRs) and bacterial artificial chromosome (BAC)-end sequences (BES-SSRs) (Odeny et al., 2007; Aruna et al., 2008; Singh et al., 2008; Saxena et al., 2010b; Songok et al., 2010; Upadhyaya et al., 2011). In summary, 23,410 gSSRs (Varshney et al., 2012a), 8,137 EST-SSRs and 6,212 BES-SSRs were developed for their utility in pigeonpea improvement (Varshney et al., 2010; Bohra et al., 2011). Many of these markers were used for genetic mapping and diversity studies. Later, with the next generation sequencing (NGS) technology, the third generation marker systems which were high throughput, efficient and more cost effective came into existence. DArT arrays comprising of 15,360 loci (Yang et al., 2006, 2011), GoldenGate platform with 768 SNPs and competitive allele-specific polymerase chain reaction (KASPar) assays for 1,616 SNPs were used efficiently in high throughput genotyping (Saxena et al., 2014). Additionally, 5,692 SFPs were also identified using six parental genotypes of three mapping populations (Saxena et al., 2011). In addition, intron spanning region (ISR) markers have also been developed from the transcriptome assembly of pigeonpea (Kudapa et al., 2012). In brief, molecular marker technologies in pigeonpea have witnessed a transition from the gel-based (RAPD) and hybridization based (RFLP, DArT, SFP) to sequencing based SSR and SNP markers.

In recent years, due to the availability of genome sequence and reduction in sequencing cost, genotyping-by-sequencing (GBS) has become a very popular approach (Elshire et al., 2011). This approach provides large number of SNPs in segregating populations that can lead to the identification of haplotypes and recombination maps. The identified haplotype blocks can then be employed as markers for mapping applications (Deschamps et al., 2012). Such markers have been developed by assessing the genome-wide sequence variations and can be effectively utilized for genetic mapping of important agronomic traits, allele mining and characterizing germplasm for genetic improvement in pigeonpea.

### Transcriptome assembly

In order to develop and enhance the genomic resources in pigeonpea, transcriptome sequencing was used as a cost effective and efficient strategy. In National Center for Biotechnology Information (NCBI), as on December 26, 2014, a total of 25,577 ESTs, are available for *C. cajan*. Apart from these, several NGS platforms were used to generate transcript reads from different tissues, developmental stages, biotic stress-challenged genotypes, especially *Fusarium* wilt (FW) and sterility mosaic disease (SMD). By using these resources, a transcriptome assembly referred to as CcTA v1 which comprised of 127,754 tentative unique sequences (TUSs) were initially developed (Dubey et al., 2011). Subsequently, this assembly was updated by analysing 128.9 and 2.19 million single-end reads from Illumina GA IIx and FLX/454 platforms to develop a comprehensive transcriptome assembly, CcTA v2 (Kudapa et al., 2012). This comprehensive assembly included four datasets and comprised of 21,434 transcript assembly contigs (TACs; Raju et al., 2010; Dubey et al., 2011; Dutta et al., 2011; Kudapa et al., 2012) and is available through the Legume Information System (LIS; http://cajca.comparative-legumes.org/). This transcriptome assembly as well as several transcriptome datasets have been used to develop functional markers (Table 3). For instance, a set of 17,113 SNPs were identified from the 128.9 million sequence reads generated using FLX/454 platforms (Dubey et al., 2011; Saxena et al., 2012). These SNPs were identified using 12 parental lines of six different mapping populations. Transcript profiling when combined with genome variants can help in identifying expression quantitative loci (eQTLs) and mapping regions with *cis*- and *trans*-effects (Holloway and Li, 2010). This is an area which could be explored and would prove useful for pigeonpea in the near future.

**Table 3:** **Genomic resources generated through transcriptome sequencing in pigeonpea**.

**Transcript sequences**	**Platform**	**Source/Genotype**	**Markers developed**			**References**
			**SSRs**	**SNPs**	**ISRs**	
5085 unigenes	Sanger	ICPL 20102, ICP 2376, ICP 7035 and TTB 7	84	102	-	Raju et al., 2010
0.12 million tentative unique sequences + 150.8 million sequence reads	Sanger FLX/454	ICPL 87119, ICPL 99050, ICPL 87091, ICPB 2049, ICPL 20096, ICPL 7035, BSMR 736, ICPL 332, TTB 7, TAT 10 and ICP 28	50,566	12,141	5,845	Dubey et al., 2011
	Illumina					
1.696 million sequence reads	FLX/454	ICPL 87119 and UPAS 120	3,771	-	-	Dutta et al., 2011
131 million sequence reads + 18353 unigenes	Illumina	-	-	-	6,284	Kudapa et al., 2012
	FLX/454					
	Sanger					
128.9 million sequence reads	Illumina	ICPL 87119, ICPL 87091, BSMR 736, TAT 10, ICP 7035, TTB 7, ICPL 332, ICPL 20096, ICPB 2049, ICPL 99050, ICP 28 and ICPW 94	-	-	17,113	Saxena et al., 2012

*SSR, Simple Sequence Repeat; SNP, Single Nucleotide Polymorphism; ISR, Intron Spanning Region*.

### Genetic maps

Genetic maps are important genomic resources for identification of molecular markers associated with traits that can be used in breeding program. In the past, due to lack of sufficient polymorphic markers along with the low level of genetic variability in pigeonpea, construction of genetic maps has been very challenging. Initially, three genetic maps were developed using the same inter-specific mapping population namely ICP 28 × ICPW 94 (Bohra et al., 2011; Yang et al., 2011). Using DArT markers, two genetic maps were generated, the maternal genetic linkage map had 122 DArT loci, while the paternal genetic map consisted of 172 paternal loci spanning a genome distance of 270.0 cM and 451.6 cM, respectively (Yang et al., 2011). In parallel, a SSR-based genetic map with a total map length of 930.90 cM was developed using 239 SSR markers with an average marker interval of 3.8 cM (Bohra et al., 2011). Two intra-specific genetic maps were also generated with 120 and 78 SSR markers spanning a distance of 534.89 cM and 466.97 cM distances, respectively (Gnanesh et al., 2011). Subsequently, a consensus genetic map was generated by merging six intra-specific genetic maps, which included two genetic maps from the previous study. The consensus map comprised of 339 SSR loci with an average inter-marker distance of 3.1 cM and spanned a distance of 1,059 cM of the genome (Bohra et al., 2012).

Very recently, due to availability of pigeonpea KASPar assay markers (PKAMs) one comprehensive genetic map has been developed for ICP 28 × ICPW 94 population. This inter-specific map is comprised of 875 PKAM loci with an average inter-marker distance of 1.11 cM (Saxena et al., 2012). Whereas, using GoldenGate SNP assays on the mapping population derived from a cross between Pusa Dwarf and HDM04-1, an intra-specific linkage map with 296 marker loci and an average marker interval of 4.95 cM has been developed (Kumawat et al., 2012).

### QTLs and candidate genes

Biotic and abiotic stresses are the major challenges to pigeonpea productivity. In India, which is the major pigeonpea growing country, biotic stresses alone could incur up to 30–100% yield loss in case of FW and up to 100% due to SMD (Sharma et al., 2012). In order to identify the genomic regions associated with resistance to these biotic stresses, various mapping populations segregating for the stresses have been developed. A significant numbers of polymorphic markers were also identified for these mapping populations (see Bohra et al., 2011; Saxena et al., 2010c). Phenotyping of FW and SMD was carried out in wilt-sick plots by screening of hundreds of lines or thousands of plants across different locations. Using these data, two RAPD markers (Kotresh et al., 2006), four SCAR markers (Prasanthi et al., 2009) and six SSR markers (Singh et al., 2013) were reported for FW resistance. In the case of SMD, six different QTLs explaining up to 24.72% phenotypic variation were identified on LG 7 and LG 9 (Gnanesh et al., 2011). Using transcript profiling a total of 118 and 33 differentially expressed genes were identified using roots and leaves of plants infected with FW and SMD, respectively (Raju et al., 2010; Dubey et al., 2011). These genes showed homology with stress-responsive genes including proline-rich protein, syringolide-induced protein, ABA-responsive protein and leucine zipper protein (Dubey et al., 2011).

As pigeonpea is known to be one of the drought tolerant crops among the grain legumes, identification of candidate genes for imparting drought tolerance will be useful across the legume crops (Narina et al., 2014). The analysis of the pigeonpea genome has identified 111 proteins which were homologous to drought-responsive universal stress proteins (Varshney et al., 2012a). For determinacy trait, *CcTFL1* gene has been identified as a likely candidate gene and validated using qRT-PCR in root tip and flower of Asha genotype (Mir et al., 2014). In addition, candidate genes such as *CcHyPRP*, *CcCDR*, *CcCYP*, *CcMT1, DLP, APB*, and *LTP1* were also identified from subtracted cDNA libraries of stress-challenged pigeonpea plants. Few stress-responsive genes were validated in Arabidopsis for conferring tolerance to drought, salinity, cold and extreme temperatures (Priyanka et al., 2010; Deeplanaik et al., 2013). In summary, a number of functional genomics approaches e.g., transcript profiling, microarrays as well as homology search have been used to identify candidate genes for various stresses (Table 4). These candidate genes once validated could be utilized for GAB in pigeonpea improvement for providing multiple stress resistance in pigeonpea.

**Table 4:** **Select set of ESTs/candidate genes identified in pigeonpea**.

**Stress**	**Source (tissue)**	**Genotype(s)**	**Transcript sequences**	**No. of DEGs/candidate genes**	**Approach**	**References**
*Fusarium* wilt	Root	ICPL 20102	**5,680** ESTs	19	Sanger	Raju et al., 2010
		ICP 2376				
Sterility mosaic Disease	Leaf	ICP 7035	**3,788** ESTs	20	Sanger	Raju et al., 2010
		TTB 7				
*Fusarium* wilt	Root	ICPB 2049	**6,673** TUSs	99	Illumina	Dubey et al., 2011
		ICPL 99050	**11,518** TUSs			
		ICPL 87119				
		ICPL 87091				
Sterility Mosaic Disease	Leaf	BSMR 736	**7,505** TUSs	13	Illumina	Dubey et al., 2011
		TAT 10	**10,497** TUSs			
		ICPL 20096	**9,402** TUSs			
		ICPL 332 TTB 7				
		ICPL 7035				
PEG/water-deficit stress	Root Leaf	ICP 8744	**75** ESTs	*CcHyPRP, CcCDR*	Homology	Priyanka et al., 2010
				*CcCYP*		
Drought, salinity and extreme temperatures		ICP 8744	**-**	*CcCYP*	Homology	Sekhar et al., 2010
Heavy metal stress		ICP 8744	**-**	*CcMT1*	Homology	Sekhar et al., 2011
Drought	Root	ICPL 8755	**724** TUSs	-	Microarray	Saxena et al., 2011
		ICPL 227				
		ICPL 151				
Drought, salt and cold	Root Leaf	ICPL 87	**-**	*CcCDR*	Homology	Tamirisa et al., 2014
		ICP 8744				
Drought	Leaf	ICPL 87119	**52** ESTs	*DLP, APB* and *LTP1*	RT-PCR	Deeplanaik et al., 2013
Determinacy	Root-tip Flower	ICPA 2039	**-**	*CcTFL1*	Homology	Mir et al., 2014
		ICPL 87118				

****ESTs***, Expressed Sequenced Tags; ***TUSs***, Tentative Unique Sequences; ***SFPs***, Single Feature Polymorphisms; ***DEG***, Differentially Expressed Genes*.

In addition to biotic and abiotic stresses, recently efforts have also been made toward mapping QTLs for some agronomic traits such as fertility restoration (Bohra et al., 2011), earliness, plant height (Kumawat et al., 2012) and determinacy (Mir et al., 2013, 2014). For instance, four major QTLs for fertility restoration namely, QTL-RF-1, QTL-RF-2, QTL-RF-3, and QTL-RF-4 explaining phenotypic variation of 14.85, 15.84, 20.89, and 24.17%, respectively were identified in three mapping populations (Bohra et al., 2012). Whereas, using genic-SNP markers, 13 QTLs were mapped for the six agronomic traits, which include plant height, number of primary branches, secondary branches, pods, days to flowering and maturity in the other mapping populations. The phenotypic variation explained by these individual QTLs ranged from 3.18 to 51.4% (Kumawat et al., 2012). Determinacy is another important adaptive trait in pigeonpea, for which six DArT and 19 SNPs have been identified using DArT arrays and GoldenGate assay in pigeonpea (Mir et al., 2013). Additionally, a novel approach known as AB-QTL (Advanced backcross-QTL; Tanksley and Nelson, 1996) can be utilized for harnessing the natural variation in a species with minimum loss of favorable alleles so as to map QTL precisely (Varshney et al., 2005; Bohra et al., 2010). In order to identify the superior alleles from the wild relative, two backcross populations are being developed using *C. cajanifolius* and *C. acutifolius* species for agronomically important traits in order to employ this promising approach (Varshney et al., 2013).

With the genome sequence information available for pigeonpea and the drastic reduction in the sequencing cost with improved sequencing chemistry have opened up a new vista for employing NGS-based approaches for identification of candidate genes/genomics region(s) underlying the trait of interest. MutMap and QTL-seq are the whole genome re-sequencing based approaches (see Varshney et al., 2014c) which could not only enhance the precision but also reduce the time considerably for trait mapping. The genomic regions thus identified would be transferred to a selected background through MAS to develop genotypes for marginal environments.

## Deployment of genomics-assisted breeding

**Figure 2:**
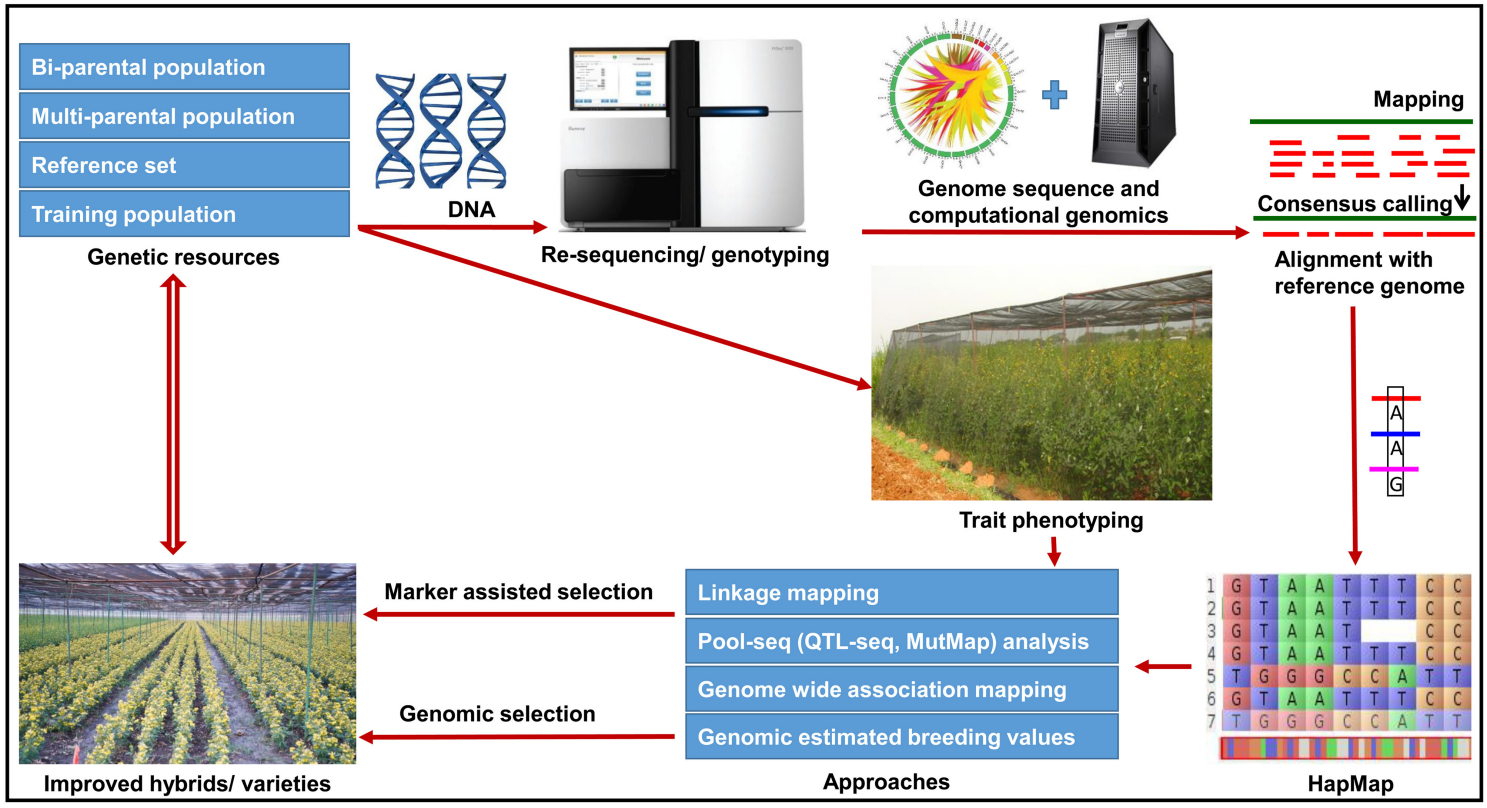
**Future directions for genomics-assisted breeding in pigeonpea**. The figure illustrates a roadmap for the utilization of various genetic and genomic resources for deploying genomics-assisted breeding for pigeonpea improvement. In order to accelerate the existing breeding efforts, the strategy has been given in the figure which will be followed in the coming years.

In pigeonpea, crop improvement programs were mainly focussed on pure line breeding (varietal improvement) and hybrid breeding. For varietal improvement many useful markers are now available for different traits as presented in Table S1. Marker-assisted backcrossing (MABC) can be utilized for simply inherited traits such as FW and SMD. MABC has been successfully applied to introgress resistance to FW and Ascochyta blight as well as drought tolerance in chickpea (Varshney et al., 2014b,c). Similarly improved lines with enhanced rust resistance have been developed in groundnut (Varshney et al., 2014d). These success stories encourage pigeonpea breeding community to deploy MABC programme to introgress resistance to diseases (FW and SMD) in susceptible cultivars as well as for pyramiding superior alleles into a single cultivar. Furthermore, trait mapping using bi-parental crosses and multi-parental crosses such as MAGIC and NAM populations are in progress which will provide additional loci for GAB in pigeonpea. A road-map summarizing different approaches to implement GAB has been presented in Figure 2.

In the context of hybrid breeding, seven cytoplasmic male sterile (CMS) systems have been developed from different wild *Cajanus* species, designated as A_1_, A_2_, A_3_, A_4_, A_5_, A_6_, and A_7_ derived from *C. sericeus* (Ariyanayagam et al., 1995), *C. scarabaeoides* (Reddy and Faris, 1981; Saxena and Kumar, 2003), *C. volubilis* (Wanjari et al., 1999), *C. cajanifolius* (Saxena et al., 2005), *C. acutifolius* (Mallikarjuna and Saxena, 2005), *C. lineatus*, (Mallikarjuna and Saxena, 2005), and *C. platycarpus* (Mallikarjuna et al., 2006), respectively. Subsequently, pigeonpea breeders developed a cytoplasmic-nuclear male sterility based hybrid system using A_4_ cytoplasm (Saxena et al., 2002; Saxena and Kumar, 2003). As a result, three A_4_ CMS-based hybrids namely ICPH 2671, ICPH 2740, and ICPH 3762 have been released successfully for cultivation in central and southern India. These hybrids produce 30–48% more yield than the popular local varieties in multi-location field trials (Saxena and Nadarajan, 2010). Although a promising technology, it often faces challenges in identifying fertility restorers, ascertaining hybrid seeds purity and maintaining three lines (CMS, maintainer and restorer lines). Having access to the genes/markers involved in cytoplasmic male sterility, fertility restoration, heterosis and purity testing will accelerate this promising technology.

In this regard, mitochondrial genomes of a CMS line (ICPA 2039), its maintainer line (ICPB 2039) and wild species (*C. cajanifolius* ICPW 29) have been sequenced. This study has revealed nine rearrangements between wild accessions and the CMS line, whereas 22 rearrangements between CMS and the maintainer line along with 34 protein coding genes in addition to presence and absence variations (PAVs) at 29 regions (Tuteja et al., 2013). These rearrangements and structural variations could cause defects in the mitochondrial respiration through production of abnormal proteins (Ma, 2013). The identification of the genes responsible for such defects can help to understand the molecular mechanisms underlying the development of CMS in pigeonpea. Based on the above mentioned mitochondrial differences between CMS and its maintainer line, molecular markers can be identified, that will be helpful in maintaining the purity of A_4_ derived CMS lines. Similarly, SSR based molecular markers kit have been developed for purity testing of pigeonpea hybrids (F_1_s derived from CMS and restorer line) (Saxena et al., 2010a; Bohra et al., 2014b). Thus, marker based genetic seed purity testing is available for pigeonpea hybrid breeding system which is relatively faster and efficient method than the conventional grow out test (GoT).

The three-line hybrid breeding system depends on maintaining CMS lines by crossing with its maintainer line and identifying perfect restorer lines, which makes the technology a bit tedious and expensive. As a result, efforts are being made to explore an alternative two-line hybrid breeding system, which requires a male sterile line that could precisely convert to fertile line and also reverts back under the influence of certain environmental factors. Toward this, a temperature-sensitive male sterile line has been identified based on the field observations in pigeonpea (Saxena, 2014). The precise characterization and evaluation of such environment-sensitive male sterile line is most critical in the development and utilization of a two-line hybrid breeding system. Apart from this, it is also important to identify the parental combinations that would give higher yields as well as better resistance to diseases. In this regard, defining heterotic groups that cater to the needs of different locations and resistance to various stresses is the need of the hour. In this context, seven heterotic pools were defined based on the specific combining ability (Saxena and Sawargaonkar, 2014). Various approaches based on genome wide markers for identification of favorable alleles in different parental genotypes would greatly aid in this aspect. In summary, above mentioned possibilities and efforts would greatly help in accelerating the pigeonpea hybrid breeding program in Asia and other regions of the semi-arid tropics.

For complex traits which are governed by many genes/minor QTLs, genome-wide selection (GWS) or genomic selection (GS) could be deployed, which is a two-step process. The first step is to calculate genomics estimated breeding values (GEVBs) from the genome-wide marker profiling and extensive trait phenotyping data on training population to define the GS models. In the second step, GEVBs are calculated in test population only on the basis of marker data (Heffner et al., 2009). This will also help in predicting the performance of a genotype which would be used in a crossing program without phenotypic evaluation over years and environments. This enhances the genetic gains by increasing the selection intensity (*i*) and reducing the length of selection cycle (*L*) according to the equation *R* = *h*^2^σ*pi*/*L* (Moose and Mumm, 2008), where *h*^2^ is the heritability and *σ p* is the phenotypic variation for the trait. In pigeonpea, GS can effectively be deployed for predicting and identifying the elite breeding materials in the initial generations by reducing the breeding cycle, which is a major constraint in the breeding program. Furthermore, a combination of MAS and GS has been recommended for crop improvement by Varshney et al. (2014a). The same could also be proposed for pigeonpea improvement programme.

### Potential challenges for deploying GAB

Besides the immense possibilities, the potential challenges of deploying GAB in pigeonpea cannot be overlooked. The first challenge in deploying GAB in pigeonpea is its long life cycle which allows producing only one generation in field conditions in the cropping season. To overcome this constraint, ample resources are required to grow large populations in controlled conditions during the off-season. The second challenge is the often cross-pollinated nature of the crop which has resulted in variable degree of heterozygosity. These factors have subsequently resulted in slowing down the crossing programs, thus lesser mapping populations were developed when compared to other crops. In addition, the cross-incompatibility barriers have led to further impeding the development of inter-specific mapping population. Apart from the above mentioned challenges, low level of genetic polymorphism, low heritable traits and photo-sensitivity pose further hindrance for GAB. Therefore, the identified marker trait associations utilizing one mapping population may not show validation in other genetic backgrounds for deploying MAS. To circumvent such situation, multi-parent mapping populations (MAGIC/NAM) are being developed, which will facilitate identification of tightly linked markers for large number of traits with high throughput genotyping and phenotyping. Furthermore, for any trait mapping experiments, high throughput and precision phenotyping is of utmost importance, which remains a serious bottleneck in case of pigeonpea. Thus, identified markers could be utilized in different genetic background after validation. Moreover, GS can be a promising futuristic approach when breeding for complex traits with low heritability. Toward applying GAB in pigeonpea, proper decision support tools needs to be made available so as to translate the information into knowledge which will ultimately be useful to the pigeonpea breeders.

## Conclusions

In the wake of global climate change scenario owing to scarcity of land and water resources, importance of drought tolerant crops like pigeonpea has been realized. Pigeonpea can play a major role in providing food security especially to the semi-arid tropics, where it can be grown under marginal environment with limited resources. However, pigeonpea productivity is severely affected by various biotic stresses such as pests and diseases. The narrow genetic base of the crop has been a major bottleneck toward implementing GAB in pigeonpea. In view of this, significant progress has been made in developing various genomic resources which include molecular markers, genetic maps and transcriptome assembly, while specialized genetic stocks such as multi-parent MAGIC and NAM populations are being generated (Figure 1). Efforts are now focussed on marker-trait association, candidate gene identification and MAS for resistance to biotic stresses (FW and SMD), tolerance to abiotic stresses (terminal drought, salinity and water-logging), agronomically important traits such as plant type and earliness. Genomics efforts are also being directed for assessing seed purity, identification of candidate genes for CMS, fertility restoration and defining heterotic pools for identifying parental combinations for accelerating the hybrid breeding programme in pigeonpea. Of late, the need to introgress the valuable traits from the wild relatives into the cultivated species has been realized and efforts have already been initiated, including AB-QTL. The availability of pigeonpea genome information has enabled many NGS-based approaches for allele mining, identification of candidate genes and genetic mapping with high resolution which has enhanced the pace, precision and efficiency of trait mapping. At present, trait-associated markers, cost-effective genotyping platforms and expertise are available for deploying GAB in pigeonpea. This has led to a paradigm shift from the development of genomic resources to deployment of GAB to hasten genetic improvement program in pigeonpea. However, there is a need to have a low cost, high-throughput and efficient field relevant phenotyping. We believe that in the coming years, extensive deployment of MAS and GS in combination or alone would be undertaken for enhancing the pigeonpea productivity.

### Conflict of interest statement

The authors declare that the research was conducted in the absence of any commercial or financial relationships that could be construed as a potential conflict of interest.
