# Supplementary material for: Genomics-assisted breeding for boosting crop improvement in pigeonpea (Cajanus cajan)
Source: Front Plant Sci. 2015 Feb 17;6:50. doi: 10.3389/fpls.2015.00050 (PMC4330709; doi:10.3389/fpls.2015.00050)
Supplement: Supplementary file 1 [file Table1.DOCX]

**Table S1: Markers identified in different studies for pigeonpea**

| **Trait** | **Marker name** | **Marker type** | **PVE** | **Forward Primer (5’-3’)** | **Reverse Primer (5’-3’)** | **Reference** |
| --- | --- | --- | --- | --- | --- | --- |
| ***Fusarium wilt* (FW)** | SCAR-1 | SCAR | - | TTCACGTCCACACT | TTCACGTCCACCAT | (Prasanthi et al., 2009) |
|  | SCAR-2 | SCAR | - | GCAAATGCACTCCAAATGAA | TTCACGTCCACCATGAAGAA |  |
|  | SCAR-3 | SCAR | - | GGTGTTTGGTGCTCACA | TTGGACCTTAGCTCCTA |  |
|  | SCAR-4 | SCAR | - | TTCACGTCCACACTTCTAGCA | GTGCCTCAAATCCTCTTCCT |  |
|  | ASSR-1 | SSR | 37.5 | GTCCGTTGAAAAACAAAGAG | CGTTTTAGGTTTCTTCTCTGC | (Singh et al., 2013) |
|  | ASSR-23 | SSR | 34.3 | CTTTCCCTTCTCTCTCAACAC | AAGCAGAAGCAGAAGCAGAG |  |
|  | ASSR-148 | SSR | 23.7 | AACCGATGCTTTCTTCTACTAC | ACTCAACGGTGCTACTCATC |  |
|  | ASSR-229 | SSR | 35.2 | ATAGTGGGACAGTAGAAAATCC | CAACTCATCTCTTGGTTCTCC |  |
|  | ASSR-363 | SSR | 56.4 | GGGAGAAGTATAAGGAGAAATG | TCACCCTTTGATAATGTTCC |  |
|  | ASSR-366 | SSR | 41.1 | CTCTGCAACTCGCTCATTTC | ACGTGATGGAGAAGATCCAAC |  |
|  | OPM03 | RAPD | - | GGGGGATGAG | - | (Kotresh et al., 2006) |
|  | OPAC11 | RAPD | - | CCTGGGTCAG | - |  |
| **Sterility mosaic disease (SMD)** | CcM1982 | SSR | - | TATCAAACCTGGCGATCACA | ATTCCGCAAACACATCACAA | (Gnanesh et al., 2011) |
|  | CcM1447 | SSR | - | CTTTCCACGGTCCAGTGAAT | TCCATTTGACATGTAAACAATGAAC |  |
|  | CcM0588 | SSR | - | AAAAACAATTATTGCGTAAGATTATCA | ACGTTAGGAGCAAAGCGTGT |  |
|  | CcM2781 | SSR | - | TCGTAGTCAAACCAAATCCCT | AAAGTGATTCATCCATAAAAAGTTTG |  |
|  | CcM2149 | SSR | - | TGTACAGGGCTGTAGGTTCG | TCATTTTGACCCTTTTTAGATTCC |  |
|  | CcM0468 | SSR | - | ATAAAAATATCCGCAACCGC | CGAAAGCAATGTCAAAGCAA |  |
|  | CcM0970 | SSR | - | TTAAAATCACATCTTACGAAACATAAA | AGGACATACGTTCCAAAATTGA |  |
|  | CcM2485 | SSR | - | TGTAGAACATGAGTTATTGAACGGA | ATTGGGTCCCAGTTTGATTG |  |
|  | CcM0416 | SSR | - | AAATTTAAAAATACTCATGTCGAAGAA | CCGTGAGTGTTAATGGACTAATATG |  |
|  | CcM2337 | SSR | - | TTGTGATAATTTTATATGTGGAAACG | GGACCCAGCAAGAAATTTGA |  |
| **Plant height** | ASNP1310 | SNP | 28 | AGATTCTCATTCACCACGAGGCAAACAAAAGATGTAGAGGTTCTTAGGGATTGTTCGAT[T/G]CGCATTCCTTGCGGGGAATTTTGGATGCTTCTCGGACCCAATGGGTGTGGAAAATCTACC | | (Kumawat et al., 2012) |
|  | ASNP2099 | SNP |  | GGTCAGTCATTGATGTCAGATAATACAAGGAACTGTATGAACACGTTTAAAAGTTAATGC[T/C]TGATTCAACTATGCCACCACTATATATTTTAAATCGTTGGTGGCAATAAATTCCATACAG | |  |
|  | ASSR-100 | SSR | 27.5 | AACTTAGACTCTGACCCATTTC | CAAGTTGAACCACTAGACACAC |  |
|  | ASSR-206 | SSR |  | GGAAGGGAAAACTTAGAGAGAG | ATATCTTCCCTACGTGTGTGTC |  |
| **Number of primary branches per plant (PB)** | ASNP1664 | SNP | 19.5 | TCACATCTCCTATTCACGCCATACTGATCTTAGTATAAAGGTGAATAATGATAGGAGCCG[A/G]GATTATACCATTCCTAATACTCCTGTTGTGAATGCGCAACCCTCAATTTTAGGGCAACAA | |  |
|  | ASSR295 | SSR |  | AATAGGTTCCAGAATGAAGC | AAGGCCTAGCTGATGTATTG |  |
|  | ASSR206 | SSR | 11.1 | GGAAGGGAAAACTTAGAGAGAG | ATATCTTCCCTACGTGTGTGTC |  |
|  | ASNP242 | SNP |  | TCCCATGTCATGGACTGCTATTTACGTTGCACTTGACAACGTGGGAATGTGGAACTTGAG[A/G]TCTGAGTTCTGGGCACGACAATACCTTGGCCAACAGTTATATTTGCGCGTTTATACATCA | |  |
| **Number of secondary branches per plant (SB)** | ASSR-100 | SSR | 10.4 | AACTTAGACTCTGACCCATTTC | CAAGTTGAACCACTAGACACAC |  |
|  | ASSR-206 | SSR |  | GGAAGGGAAAACTTAGAGAGAG | ATATCTTCCCTACGTGTGTGTC |  |
| **Number of pods per plant (PD)** | ASNP64 | SNP | 3.2 | AATCACCTACGTTCACATGCAGATCATCATTTTCATCTTTTTTGGTGGAACCAGTCAC[A/T]CCATTTCTAGTTTCTGGTGGGTTTTCATTCAAGAGTGTGGGCGATTCCACTTGTTTGGAA | |  |
|  | ASNP882 | SNP |  | CACAATCTTTAAAATATGGATAGAATCTAGAATCTTCAAATATTTCTGAAGGAACACCA[A/G]GGCCAGGAGGCTGAAAGAAATCTAGTGAAATTGACATTATAGCATTCAAAACATTGTTAA | |  |
|  | ASNP2099 | SNP | 16.5 | GGTCAGTCATTGATGTCAGATAATACAAGGAACTGTATGAACACGTTTAAAAGTTAATGC[T/C]TGATTCAACTATGCCACCACTATATATTTTAAATCGTTGGTGGCAATAAATTCCATACAG | |  |
|  | ASNP269 | SNP |  | AAAGCAGAGCAGAAGTGAGAAAAAGAGCCGAAAAGCAATGTTGAAGCTGGGATTGAAACC[A/T]GTTACCGGCGTTAGTAGGGTCACAATCAAGAGAACAAAAAATATTCTTTTCTTCATC | |  |
|  | ASSR-100 | SSR | 18.9 | AACTTAGACTCTGACCCATTTC | CAAGTTGAACCACTAGACACAC |  |
|  | ASSR-206 | SSR |  | GGAAGGGAAAACTTAGAGAGAG | ATATCTTCCCTACGTGTGTGTC |  |
| **Days to flowering (FL)** | ASNP1310 | SNP | 51.4 | AGATTCTCATTCACCACGAGGCAAACAAAAGATGTAGAGGTTCTTAGGGATTGTTCGAT[T/G]CGCATTCCTTGCGGGGAATTTTGGATGCTTCTCGGACCCAATGGGTGTGGAAAATCTACC | |  |
|  | ASNP2099 | SNP |  | GGTCAGTCATTGATGTCAGATAATACAAGGAACTGTATGAACACGTTTAAAAGTTAATGC[T/C]TGATTCAACTATGCCACCACTATATATTTTAAATCGTTGGTGGCAATAAATTCCATACAG | |  |
|  | ASSR-100 | SSR | - - 1. 7 | AACTTAGACTCTGACCCATTTC | CAAGTTGAACCACTAGACACAC |  |
|  | ASSR-206 | SSR |  | GGAAGGGAAAACTTAGAGAGAG | ATATCTTCCCTACGTGTGTGTC |  |
| **Days to maturity (MT)** | ASNP2099 | SNP | 22.6 | GGTCAGTCATTGATGTCAGATAATACAAGGAACTGTATGAACACGTTTAAAAGTTAATGC[T/C]TGATTCAACTATGCCACCACTATATATTTTAAATCGTTGGTGGCAATAAATTCCATACAG | |  |
|  | ASNP269 | SNP |  | AAAGCAGAGCAGAAGTGAGAAAAAGAGCCGAAAAGCAATGTTGAAGCTGGGATTGAAACC[A/T]GTTACCGGCGTTAGTAGGGTCACAATCAAGAGAACAAAAAATATTCTTTTCTTCATC | |  |
|  | ASSR-100 | SSR | 25.9 | AACTTAGACTCTGACCCATTTC | CAAGTTGAACCACTAGACACAC |  |
|  | ASSR-206 | SSR |  | GGAAGGGAAAACTTAGAGAGAG | ATATCTTCCCTACGTGTGTGTC |  |
|  | ASNP2262 | SNP | 3.2 | GCCTGTGGCTGACACAAGCCTGAAACGCTAGAACCTTCACCTCCATCATTAAGATTGAGT[A/G]TCATATTGTCATCCTGTGGTTTCTCAGAAAATTTCATTGCCTTGAGAACATAATTGTCGT | |  |
|  | ASNP2402 | SNP |  | ATTTTCTCAATTTTTGCATTGGTTTGAGATATCTTTTTCTCTAGCGCTTCAGGAGTTAAG[T/C]TCTTTTTTCTCTTTCCATCTGAACTCTTTGAAGGGGGCTTTCCTTTCCTTGCCCTGTCTA | |  |
| **Fertility restoration (FR)** | CcM1522 | SSR | 14.9 | TGAAATGAGTCTACCAAAGCTTACTC | TTGATCTATGCACCAAGAACTTTT | (Bohra et al., 2012) |
|  | CcM1821 | SSR |  | AAACGCAAAAGGTAAACATGC | TGATATTTGTCAAACTTCTGAAGGA |  |
|  | CcM0047 | SSR | 15.8 | TGTCTTTTGGATGAAAGTAGGGA | GTTGGGGATGGGAAGAGAAT |  |
|  | CcM2332 | SSR |  | TCCATTTTTCTTCTGCGGTG | TGCTATCAATCCAAAAACACAAA |  |
|  | CcM2542 | SSR | 20.9 | AACCCGTAAATAATTCTTCTCACA | AAATTCGTTGGAATCTTTGCT |  |
|  | CcM1277 | SSR |  | TACCTTTGGAGGCTTTGGTG | TTGCGACAACCCTGTCAATA |  |
|  | CcM0374 | SSR | 24.2 | GAACCGTCTTAAAATTTCTCATTT | CAATGGCACATTGTCAAAAA |  |
|  | CcM1506 | SSR |  | TGTTTTTGCAAAGGTTTCCC | CAACACAATGAAAAAGTAAACATCA |  |
| **Determinacy** | TFL1_PCR_C | AS | - | GGTACTCATTATACCATCATTTGAG | GCATTGAAGTAGACAGCAGC | (Mir et al., 2014) |
|  | TFL1_PCR_A | AS | - | GGATTCTTTTAACAACTCAACAAAAA | - |  |
|  | TFL1_PCR_T | AS | - | GTACTTTTAAATGATTATCTTAAAAA | - |  |

**PVE:** Phenotypic variance explained**; SCAR:** Sequenced characterized amplified region**; SSR:** Simple sequence repeat**; RAPD:** Random amplified polymorphic DNA**; SNP:** Single nucleotide polymorphism**; AS:** Allele specific
